# Undifferentiated Pleomorphic Sarcoma of Pancreas: A Case Report and Review of the Literature for the Last Updates

**DOI:** 10.1155/2018/1510759

**Published:** 2018-05-31

**Authors:** Behnam Sanei, Amirhosein Kefayat, Melika Samadi, Parvin Goli, Mohammad Hossein Sanei, Fatemeh Ghahremani, Alireza Amouheidari

**Affiliations:** ^1^Department of Surgery, Isfahan University of Medical Sciences, Isfahan, Iran; ^2^Cancer Prevention Research Center, Isfahan University of Medical Sciences, Isfahan, Iran; ^3^Department of Oncology, Seyed Al-Shohada Hospital, Isfahan University of Medical Sciences, Isfahan, Iran; ^4^Department of Pathology, Isfahan University of Medical Sciences, Isfahan, Iran; ^5^Department of Medical Physics, School of Medicine, Isfahan University of Medical Sciences, Isfahan, Iran; ^6^Department of Radiation Oncology, Isfahan Milad Hospital, Isfahan, Iran

## Abstract

The most prevalent type of soft tissue sarcoma is undifferentiated pleomorphic sarcoma (UPS) or previously known as malignant fibrous histiocytoma. It accounts over 20% of all soft tissue sarcomas and occurs most frequently in the extremities, trunk, and retroperitoneum. However, it has been rarely observed in the digestive system. Pancreas sarcoma represents less than 1% of all pancreatic tumors, and primary UPS of the pancreas is even rarer. It exhibits high recurrence and poor prognosis. In this case, a 72-year-old woman with a UPS tumor which was located in the pancreas head and neck without adhesion to the retroperitoneum will be discussed.

## 1. Introduction

Undifferentiated pleomorphic sarcoma (UPS) or previously known malignant fibrous histiocytoma is the most common type of soft tissue sarcoma of middle and late adulthood [1–3]. It is more prevalent in men than in women and usually occurs in the extremities and retroperitoneum. However, digestive organ involvement is extremely rare [4]. UPS of the pancreas is one of the rarest primary nonepithelial tumors in the pancreas which is highly aggressive with poor prognosis [5, 6]. The diagnosis is usually difficult and commonly achieved after surgery. Herein, we report a 72-year-old woman with abdominal pain for about 1 year who underwent total pancreatectomy, and the postoperative pathological examinations revealed UPS.

## 2. Case Report

**Figure 1:**
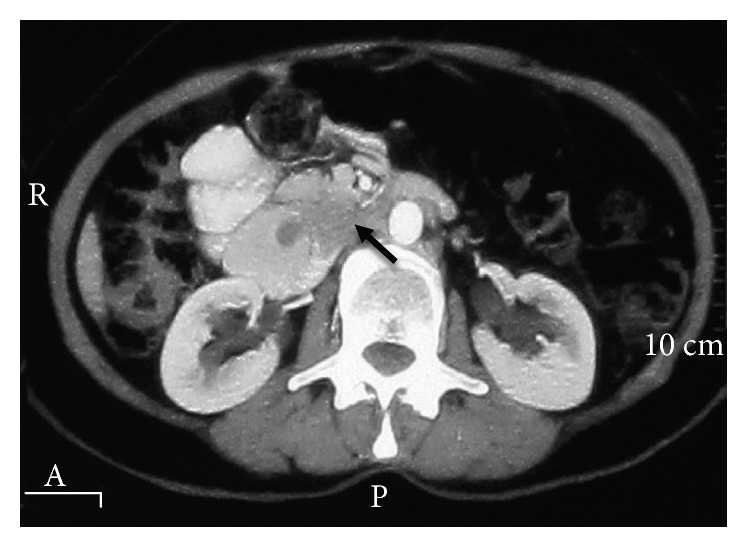
The preoperative abdominal CT scan exhibited a hypodense lesion in the pancreas head and neck (the black arrow indicates the mass).

**Figure 2:**
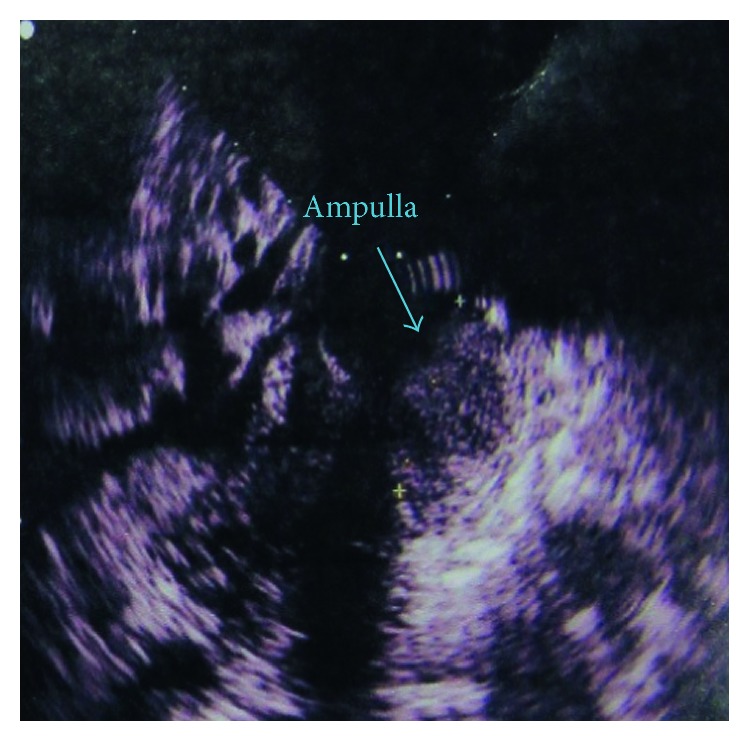
Endosonographic imaging exhibited a hypoechoic lesion at the ampulla.

A 72-year-old woman was referred to our hospital with pancreatic head and neck carcinoma for more evaluations and surgical operation. She had been suffering from abdominal pain for about 1 year, and the pain has become more intense in the last 6 months. The pain was postprandial and localized to the right upper abdomen. She has become icteric with generalized spread plus itching since past 2 months. The patient had no history of alcohol consumption or smoking. The patient was referred to us with a highly probable diagnosis of pancreatic head and neck carcinoma to be consulted for surgery. Also, a plastic stent was implanted for her in the previous health center due to intra- and extrahepatic duct dilation for the relief of patient symptoms and signs. After admission to our hospital, she was evaluated by abdominal computed tomography (CT), endosonographic imaging, and diagnostic ampullary biopsy. The abdominal CT scan with contrast exhibited an 18 mm × 20 mm hypodense mass at the head and neck of the pancreas (Figure 1). Also, endosonographic imaging was performed to rule out periampullary lesions (Figure 2). A 20 mm × 19 mm lesion was seen in the pancreatic head and neck region, and the main pancreatic duct was slightly dilated in the body of the pancreas. Moreover, the common bile duct (CBD) was distally thickened and contained sludge. Subsequently, diagnostic biopsy for pathological assessment was done. The biopsy revealed an irregular gray-creamy soft tissue which had undifferentiated malignant tumor features at microscopic evaluations. During the Whipple procedure, after cutting the neck of the pancreas in the left side of the portal vein, the frozen section revealed more involvement of pancreatic tissue. Although additional 2 cm was resected, the pancreas residue still had tumor involvement, macroscopically. Therefore, the patient was undergone total pancreatectomy.

**Figure 3:**
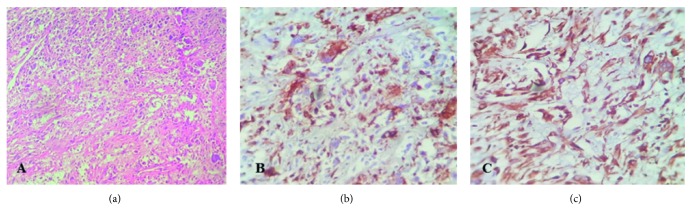
(a) Microscopic sections reveal the proliferation of mesenchymal cells in a storiform pattern. Cells consist of two populations including spindle fibroblast-like and pleomorphic cells. (b) The giant cells are positive for CD68 marker. (c) The cells exhibit the diffuse positive reaction to vimentin.

Postoperative pathological studies were established and indicated a tumor with the greatest dimension of 4 cm which was extended to the duodenum. Invasion of the venous, lymphatic vessels and perineural sites was seen; however, no exact evidence of distant metastasis was found. The periampullary occlusion had hindered the bile flow over time, leading to chronic cholecystitis and pancreatitis which was confirmed by histopathological assessments. The hematoxylin and eosin staining revealed the presence of two cellular populations including spindle fibroblast-like and pleomorphic cells within the tumor. In addition, the proliferation pattern of the mesenchymal cells was storiform (Figure 3(a)). There was not any well-differentiated component in the tumor tissue or adjacent tissue. Overall, the pathologic stage II A was assigned to the tumor. The immunohistochemical staining was performed, and the tumor was positive for CD68, lysozyme, alpha 1-antichymotrypsin, and vimentin (Figure 3). Also, it was negative for S-100P, cytokeratin, epithelial membrane antigen, desmin, CD34, smooth muscle antigen, MDM2, and CDK4. Therefore, the tumor diagnosis was compatible with UPS. The tumor Ki-67 expression was more than 30%. To evaluate metastasis occurrence, contrast-enhanced thoracic high-resolution CT scan and multidetector CT scan of abdominopelvic were done at 6, 12, 18, 24, and 36 months after operation, and no evidence of metastasis was detected. Insulin and Creon were started after surgery for long life. The further follow-up investigations were done by periodic CT scan and ultrasonic imaging. Fortunately, she was disease-free during 5-year follow-up and tolerated total pancreatectomy, well.

## 3. Discussion

There are currently two hypotheses regarding the etiology of UPS. The first one suggests these tumors do not actually represent a type of cancer but rather a common “morphologic pattern” shared by many neoplasms. This common morphologic pattern is probable to be the fate of a final common malignant progression pathway as tumors become progressively more undifferentiated. Therefore, UPS can originate from not only sarcomas but also carcinomas. The second less common one hypothesizes that undifferentiated sarcomas are originated from malignant transformation of mesenchymal stem cells which do not express differentiation markers from the beginning [7, 8].

Primary UPS of the pancreas as a subtype of pancreatic sarcoma is extremely rare [4, 9]. It usually appears as a large mass and its mean diameter is within 4–35 cm range with high recurrence rate and poor prognosis [6, 10]. Clinical presentations are usually epigastric pain, nausea, and vomiting. Some patients may present with weight loss and abdominal mass. According to the location of tumor at pancreas, jaundice is probable [11, 12]. Primary UPS of the pancreas represents a large, heterogeneous, low-attenuation density or multinodular mass with probable intratumoral calcification and a large amount of necrosis in CT scan imaginings. Contrast modalities reveal a nonhomogeneously enhancing mass within the pancreas by enhancing peripheral pseudocapsular mass [5, 13]. Magnetic resonance imaging (MRI) is even more helpful than CT scan, but features are nonspecific for UPS. Primary UPS has high signal intensity on T2-weighted MRI. Also, nonhomogeneous isosignal intensity expression can distinguish it from surrounding tissues [14]. Microscopic findings will narrow our spectrum of diagnosis. UPS usually consists of spindle fibroblast-like and round histiocyte-like cells. However, immunohistochemistry (IHC) and genetic test are really necessary for final diagnosis.

Immunohistochemical exams can exclude other mimic tumors and finalize the UPS diagnosis. The absence of characteristic epithelial markers such as desmin, keratin, S-100P, alpha-fetoprotein, and CEA can exclude the epithelial origin. In addition, UPS exhibits characteristic that have strong reactions to vimentin, alpha 1-antichymotrypsin, CD68, and lysozyme as this case did [15, 16]. Most UPS diagnosed tumors may be rather a poorly differentiated sarcoma, specially dedifferentiated liposarcomas. This is due to technical limitations, such as tumor sampling, and technical investigations such as ultrastructural study, immunohistochemistry, and molecular analysis for identifying any evidence of differentiation [17]. The dedifferentiated liposarcomas are characterized by the ring or giant-marker chromosomes derived from the q13–15 region of chromosome 12 which cause amplification of MDM2, CDK4, SAS, and GLI genes [8, 18–20]. Therefore, many papers have introduced the immunohistochemistry assessment of the MDM2 and CDK4 marker expression as a good key to exclude dedifferentiated liposarcoma from the probable diagnoses [21]. In our case, expression of these markers was negative.

The UPS genetic aspects are hard to assess because of the shifting diagnostic criteria used throughout the recent years. In general, their karyotypes have high complexity, with extensive intratumoral heterogeneity. Considerable percent of cases exhibit triploid or tetraploid chromosomes, and a few cases are near-haploid [22, 23]. Although no specific structural or numerical aberrations have emerged, telomeric associations, ring chromosomes, and/or dicentric chromosomes are common. The comparative genomic hybridizations have demonstrated many genomic imbalances like loss of 2p24-pter and 2q32-qter, and chromosomes 11, 13, and 16, as well as the gain of 7p15-pter, 7q32, and 1p31 [24–26]. In addition, it seems 12q13-15 region of chromosome plays a critical role in the development of UPS. Therefore, SAS, MDM2, CDK4, DDIT3, and HMGIC have all been reported to be amplified in MFH [27–29]. Alterations of TP53, RB1, and CDKN2A have been suggested to participate in rising of UPS. This alteration includes mutation and/or deletion, but no apparent relationship with clinical outcome has yet been announced [23, 30, 31].

The origin of the term “malignant fibrous histiocytoma (MFH)” dates back to the early 1960s. Extraction and culturing of cells from some tumors exhibited a storiform pattern which contained pleomorphic and giant cells with phagocytosis-like movements, these features seemed reminiscent of histiocytes. Upon further growth, these cells became elongated and assumed a fibroblastic appearance. Based on these observations, the hypothesis that pleomorphic soft tissue tumors arose from the fibroblastic transformation of histiocytes was suggested [32]. Therefore, the term “malignant fibrous histiocytoma” was chosen. Due to the vast range of histological appearance at the MFH diagnosed tumors, they were further subdivided into (1) storiform-pleomorphic, (2) myxoid (myxofibrosarcoma), (3) giant cell, (4) inflammatory, and (5) angiomatoid [8]. Revision of the previously categorized MFH tumors by available modern immunohistochemical techniques revealed only 13–27% concordance rate over MFH diagnosis. According to the 2002 WHO classification, the term “undifferentiated pleomorphic sarcoma” was replaced by the old MFH terminology. In addition, myxoid and angiomatoid MFH were no longer subtype of MFH and relocation to other categories. The existence of giant cell and inflammatory MFHs as distinct entities was also questioned. Undifferentiated pleomorphic sarcoma with giant cells was named after conditions in which no evidence of differentiation is found plus presence of giant cells. Also, inflammatory MFH can be made only if all markers of a mesenchymal lineage were negative. In the 2002 WHO classification, this term was renamed to undifferentiated pleomorphic sarcoma with prominent inflammation [7, 23, 33–36]. In the 2013 WHO classification, undifferentiated pleomorphic sarcoma and its subtypes were renamed “undifferentiated sarcoma” and reclassified under the undifferentiated/unclassified sarcomas. These tumors showed lack of distinct clinical or morphological characteristics that would otherwise place them under specific types of sarcomas. The new subtypes include (1) undifferentiated spindle cell sarcoma, (2) undifferentiated round cell sarcoma, (3) undifferentiated epithelioid sarcoma, (4) undifferentiated pleomorphic sarcoma, and (5) undifferentiated sarcoma NOS. Genetic subgroups can be really helpful in this family, and this important work is ongoing [37–40].

To the best of our knowledge, 21 cases of pancreas UPS have been reported in the English-language literature up to now (Table 1). They consisted of 15 men and 6 women. The mean age at diagnosis was 55 years, with a range from 22 to 77 years. Three histologic types were observed among the 21 cases: 14 storiform-pleomorphic (or pleomorphic), 3 giant cells, and 4 myxoid. In 11 cases, tumors were located in the body and/or tail of the pancreas and left pancreatectomy and splenectomy were performed. In 9 cases, the tumor was located in the pancreas head and pancreaticoduodenectomy was chosen as the choice treatment. One patient underwent enucleation of the tumor that was located in the uncinate lobe of the pancreas. All disease tumors are categorized based on the previous classification.

Of these 21 UPS cases, one patient died of perioperative complications. Five patients died of the main disease 5, 7, 11, 12, and 24 months after surgery. 2 patients have not been followed up, and 13 patients were alive without evidence of recurrence, with a median follow-up period of 15 months (mean: 17.5 months and range: 2 to 48 months). Only 8 patients survived for more than one year and just 3 for more than two years. It is apparent that exact conclusions can not be made from such a diverse and limited number of cases that lack appropriate follow-up data. Nonetheless, this evaluation of patients with primary pancreas UPS suggests a poor prognosis for long-term survival. This can be explained by the high malignant potential as well as delayed diagnosis. Furthermore, from 21 introduced UPS cases, only 1 case represented evidence of calcification [43] and 7 cases had a necrotic tumor with internal hemorrhage. Based on these observations, it would appear that calcification or necrosis and hemorrhage are not always helpful in differentiating UPS from other tumors in the pancreas.

None of the 21 patients with pancreas UPS had pulmonary, hepatic, or other distant metastasis at the time of surgery. Only one case was reported with lymph node metastases [13]. Also, one case recurrence was reported 11 months after surgery with hepatic and pulmonary metastasis. The patient underwent a multidisciplinary treatment of chemotherapy, radiotherapy, and a right hepatectomy combined with intraoperative radiofrequency ablation. Under multidisciplinary treatment, the patient fully recovered and remained disease-free for about 25 months after tumor recurrence.

In summary, our knowledge about UPS of the pancreas is really inadequate especially because of its rarity. More cases with definitely follow-ups are needed to perform a representative clinical analysis.

## Figures and Tables

**Table 1:** Cases of primary UPS of the pancreas in English-language literature.

Authors (ref. no.)	Year	Age	Sex	Histologic type	Location	Treatment	Follow-up (months)
Margueles et al. [12]	1976	22	F	Myxoid	Head	Pancreaticoduodenectomy	17, NED
Ishiguchi [41]	1986	44	M	Pleomorphic	Body-tail	Left pancreatectomy, splenectomy	15, NED
Hasegawa et al. [42]	1987	44	M	Giant cell	Body-tail	Left pancreatectomy, splenectomy	7, NED
Garvey et al. [43]	1989	77	M	Storiform-pleomorphic	Uncinate lobe	Enucleation	48, NED
Pascal et al. [44]	1989	39	M	Storiform-pleomorphic	Head	Pancreaticoduodenectomy	0, DOC
Suster et al. [45]	1989	71	M	Giant cell	Head	Pancreaticoduodenectomy	NF
Allen et al. [46]	1990	46	M	Storiform-pleomorphic	Body-tail, local invasion	80% pancreatectomy, splenectomy, subtotal gastrectomy	5, DOD
Filippini et al. [47]	1992	50	F	Myxoid	Body-tail	Left pancreatectomy, splenectomy	NF
Tsujimura et al. [48]	1992	43	F	Storiform-pleomorphic	Tail	Pancreatectomy, splenectomy	5, NED
Ben [49]	1993	72	M	Storiform-pleomorphic	Body-tail	Left pancreatectomy, splenectomy	12, DOD
Balén [50]	1993	37	M	Pleomorphic	Body-tail	Extended left pancreatectomy	7, DOD
Haba et al. [51]	1996	70	M	Storiform-pleomorphic	Head	Pancreaticoduodenectomy	22, NED
Bastian et al. [13]	1999	67	M	Storiform-pleomorphic	Body	Left pancreatectomy, splenectomy, transverse colectomy, subtotal gastrectomy	34, NED
Liu et al. [5]	1999	27	F	Myxoid	Body-tail	Left pancreatectomy, splenectomy	6, NED
Mai et al. [52]	2002	71	F	Giant cell	Head	Pancreaticoduodenectomy	24, DOD
Darvishian et al. [53]	2002	74	M	Storiform-pleomorphic	Head	Pancreaticoduodenectomy	4, NED
Akatsu et al. [16]	2005	67	M	Storiform-pleomorphic	Body-tail	Left pancreatectomy, splenectomy, transverse colectomy, total gastrectomy	35, NED
Yu et al. [54]	2008	67	M	Storiform-pleomorphic	Head	Pancreaticoduodenectomy	11, DOD
Jarry et al. [6]	2010	45	M	Storiform-pleomorphic	Head	Multidisciplinary treatment	11, NED
Gupta et al. [55]	2012	52	M	Myxoid	Body-tail	Distal pancreatectomy, splenectomy	2, NED
Current case	2016	72	F	Pleomorphic	Head and neck	Pancreaticoduodenectomy	22, NED

M: male; F: female; NF: no follow-up; NED: no evidence of disease; DOD: dead of disease; DOC: dead of perioperative complications.
